# A Nomogram for Preoperative Prediction of the Risk of Lymph Node Metastasis in Patients with Epithelial Ovarian Cancer

**DOI:** 10.3390/curroncol30030250

**Published:** 2023-03-13

**Authors:** Huiling Xiang, Fan Yang, Xiaojing Zheng, Baoyue Pan, Mingxiu Ju, Shijie Xu, Min Zheng

**Affiliations:** Department of Gynecologic Oncology, State Key Laboratory of Oncology in South China, Collaborative Innovation Center of Cancer Medicine, Sun Yat-sen University Cancer Center, Guangzhou 510060, China

**Keywords:** epithelial ovarian cancer, lymph node metastasis, computed tomography, nomogram

## Abstract

Objective: To develop a nomogram for predicting lymph node metastasis (LNM) in patients with epithelial ovarian cancer (EOC). Methods: Between December 2012 and August 2022, patients with EOC who received computed tomography (CT) and serological examinations and were treated with upfront staging or debulking surgery were included. Systematic pelvic and para-aortic lymphadenectomy was performed in all patients. Univariate and multivariate analysis was used to identify significant risk factors associated with LNM. A nomogram was then constructed to assess the risk of LNM, which was evaluated with respect to its area under the receiver operating characteristic curve (AUC), calibration, and clinical usefulness. Results: Of 212 patients enrolled in this study, 78 (36.8%) had positive LNs. The nomogram integrating CT-reported LN status, child-bearing status, tumour laterality, and stage showed good calibration and discrimination with an AUC of 0.775, significantly improving performance over the CT results (0.699, *p* = 0.0002) with a net reclassification improvement of 0.593 (*p* < 0.001) and integrated discrimination improvement of 0.054 (*p* < 0.001). The decision curve analysis showed the nomogram was of clinical use. Conclusions: A nomogram was constructed and internally validated, which may act as a decision aid in patients with EOC being considered for systemic lymphadenectomy.

## 1. Introduction

Ovarian cancer continues to be the most lethal gynaecological malignancy, with an estimated 239,000 new cases and 152,000 deaths reported worldwide in 2018 [1]. Nearly 90% of all ovarian malignancies constitute epithelial ovarian cancer (EOC), a group of heterogeneous diseases [2,3]. Approximately 75% of all patients with EOC are diagnosed during the late stages, with a relative 5-year survival rate of 29%, compared with the survival rate of 92% among those with early-stage disease [4]. Cytoreductive surgery for achieving no gross residual tumour (R0) followed by platinum–taxane chemotherapy remains the gold standard for the primary treatment of EOC [5]. EOC commonly spreads via the lymphatic system. Systemic lymphadenectomy is the standard procedure for all patients diagnosed with ovarian cancer; this method not only removes metastatic LNs with the goal of R0 resection but also helps to accurately assess the disease stage. Pelvic LN resection needs to remove common iliac, external iliac, internal iliac and obturator LNs, and para-aortic LN resection included removal of LNs along the aorta and vena cava to the level of the renal vein. Such complicate interventions are bound to cause postoperative complications, such as a longer surgery time, prolonged hospital stays, higher rates of infection that require treatment with antibiotics, repeat laparotomy for complications, lymphocyst development, lower-limb lymphedema, and associated pain as well as limited mobility, all of which adversely affect the quality of life [6,7].

As reported previously [8,9], in patients with early-stage disease (International Federation of Obstetrics and Gynaecology (FIGO) stages I and II), the mean incidence of LN metastases (LNM) was 14.2% (range, 6.1–29.6%), suggesting that overtreatment occurs in at least 80% of cases. In contrast, 44–53% of patients with late-stage disease have LNM [6,10]. The prospective Lymphadenectomy in Ovarian Neoplasms (LION) trial demonstrated that patients with advanced EOC who were clinically node-negative and had undergone R0 resection did not benefit from systemic lymphadenectomy; instead, they experienced a higher percentage of complications and mortality than those who did not undergo systemic lymphadenectomy [7]. Therefore, determining an accurate preoperative method for stratifying patients with EOC based on their risk of LNM will be of great significance to the era of precision medicine.

Imaging techniques are commonly used during preoperative ovarian cancer evaluation. Positron emission tomography was shown to have a high sensitivity and specificity for identifying metastatic LNs, as confirmed by the high fluorine 18 (^18^F) fluorodeoxyglucose (FDG) uptake of malignant tumour cells [11,12,13,14]. However, this technique is not always feasible as a routine diagnostic tool owing to its high cost. Computed tomography (CT) remains the first-line imaging method owing to its wide availability, fast data acquisition, and high image resolution [15]. An LN with a short-axis diameter over 10 mm was considered malignant in previous studies [16,17,18]. However, the studies were limited by the low sensitivities (35–50%) and areas under the receiver operating characteristic (ROC) curves (AUCs; 0.57–0.717) [16,17,18,19]. A poor diagnostic performance like this can lead to a high proportion of patients being under-staged or over-staged. A more accurate, non-invasive diagnostic tool is thus needed to identify patients with EOC with a high risk of LNM.

Several clinical parameters are associated with LNM in patients with ovarian cancer. Cancer antigen-125 (CA125), a reliable biomarker, is widely used in ovarian cancer screening, diagnosis, and treatment monitoring [20]. CA125 was also identified as an independent predictor of LN involvement [21,22]. Moreover, the presence of bilateral tumours was suggestive of the presence of LNM [23]. Herein, the purpose of this study is to build a nomogram incorporating the CT-reported LN status and other independent risk factors to predict the possibility of LN involvement in patients with EOC.

## 2. Methods

### 2.1. Participants

This study was performed in line with the principles of the Declaration of Helsinki. The Institutional Review Board of Sun Yat-Sen University Cancer Centre approved this study and waived the requirement for informed consent due to the retrospective nature. Consecutive patients with newly diagnosed EOC who underwent preoperative CT scanning and were treated with comprehensive staging surgery or cytoreductive surgery between December 2012 and August 2022 in our centre were enrolled in this study. Patients were included if (i) they had histologically proven EOC and had undergone pelvic and para-aortic lymphadenectomy (≥10 nodes); (ii) the time interval between the preoperative examination and surgery was <21 days; and (iii) complete clinicopathological information was available. However, patients were excluded if (i) they had received neoadjuvant chemotherapy or incomplete surgery; (ii) had epithelial cancer of unknown origin; and (iii) had not undergone lymphadenectomy or only pelvic/para-aortic lymphadenectomy. In addition, patients with mucinous ovarian carcinoma were also excluded, as such patients have a low risk of LNM [24,25]. FIGO 2014 was used for tumour staging [9]. The patients’ clinicopathological parameters, including age, FIGO stage, fertility status, laterality, presence of ascites, laboratory test results, LNM, and histological subtypes, were reviewed. The threshold values for CA125, cancer antigen-153, and cancer antigen-199 were set as 35, 25, and 35 U/mL, respectively. A total of 212 patients were finally included in the analysis.

Each patient underwent CT scanning, which was performed using multidetector row CT systems, mainly the Aquilion IQon Spectral CT (Philips Healthcare, Best, The Netherlands), Discovery 750 HD (GE Healthcare, Milwaukee, WI, USA), Somatom Force (Siemens Healthcare GmbH, Erlangen, Germany), and uCT780 (United Imaging Healthcare, Shanghai, China) systems. The acquisition parameters were set as follows: tube voltage, 120 kVp; tube current, 134–426 mA; matrix, 512 × 512; slice thickness, 5 mm, and slice interval, 5 mm (Table S1). Both non-enhanced and contrast-enhanced CT images were acquired from every patient. After routine non-enhanced CT and the intravenous administration of 2.0 mL/kg of iodinated contrast material at a rate of 2.5–3.0 mL/s, arterial, venous, and delayed-phase contrast-enhanced CT scanning was performed after 25–35 s, 60–70 s, and 3–5 min delays, respectively. Every CT scan was reviewed by at least two radiologists. Nodes with a short-axis diameter > 10 mm on CT were considered suspicious. 

### 2.2. Statistical Analysis

All of the statistical tests were performed using R software (https://www.r-project.org, accessed on 20 December 2022), SPSS 27.0 software (IBM Corporation, Armonk, NY, USA) and GraphPad Prism version 7.0 (GraphPad Software, San Diego, CA, USA). Categorical variables were expressed as frequencies and percentages, whereas continuous variables were expressed as means ± standard deviations (SDs). Univariate logistic regression analysis was performed to assess the associations between LNM and the preoperative clinical characteristics; variables with *p* values < 0.2 were subjected to multivariable logistic regression analysis. Odds ratios (ORs) and 95% confidence intervals (CIs) were also calculated. Covariates with *p* values < 0.1 were used in the nomogram construction. 

To evaluate the discrimination of the nomogram, a ROC curve was drawn, and the area under the ROC curve (AUC) was calculated. Internal validation of the AUC of the constructed logistic regression model was performed by bootstrapping with 1000 resamples. DeLong’s test was used to assess the differences in the nomogram- and CT-related AUCs, and the nomogram with and without CA125 [26]. Moreover, to evaluate whether the new nomogram could improve the predictive performance of the CT results, we calculated the net reclassification improvement (NRI) and integrated discrimination improvement (IDI) [27,28]. A calibration curve was plotted to assess the agreement between the predictive probability and actual LN status, and the agreement was further assessed using the Hosmer–Lemeshow test (*p* > 0.05 indicated good consistency). To determine the clinical utility of the nomogram, a decision curve analysis (DCA) curve was constructed by plotting the net benefit against the threshold probability [29]. 

## 3. Results

### 3.1. Baseline Information

The detailed clinicopathological characteristics of the 212 patients are presented in Table 1. The mean patient age was 50.8 (SD: 9.4) years. Of the 212 patients, 124 (58.5%) had high-grade serous cancer, and the remaining 88 primarily had clear cell carcinoma (n = 34, 16.0%), endometrial carcinoma (n = 25, 11.8%), low-grade serous cancer (n = 9, 4.2%), mixed carcinoma (n = 5, 2.4%), and other types of EOC (n = 15, 7.1%). The average number of resected LNs was 37.2 (SD: 15.5) (28.5 (SD: 11.7) pelvic and 8.7 (SD: 7.6) para-aortic LNs). The incidence of LNM was 5.2% (11/212) for the pelvic LNs, 10.8% (23/212) for the para-aortic LNs, and 20.8% (44/212) for both the pelvic and para-aortic LNs. For patients with LN involvement, the average numbers of metastatic LNs, pelvic nodes, and para-aortic nodes were 10.1 (SD: 13.6), 5.8 (SD: 8.7), and 4.3 (SD: 6.2), respectively.

### 3.2. Univariate and Multivariate Analyses of LNM-Related Factors in Ovarian Cancer 

**Figure 1:**
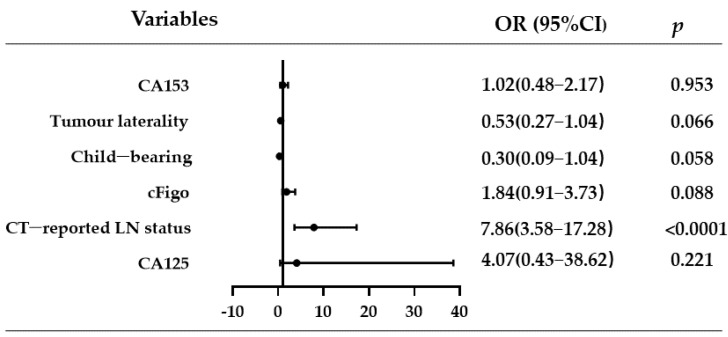
Risk factors in the multivariate regression analysis. Abbreviations: CT, computed tomography; LN, lymph node; cFigo, clinical International Federation of Obstetrics and Gynaecology; OR, odds ratio; CI, confidence interval.

The results of the univariate and multivariate analyses of preoperative factors predicting LN metastasis are shown in Table 2. The results of univariate regression analysis revealed that CT-reported LNM (*p* < 0.0001), an advanced clinical FIGO stage (cFigo) (*p* < 0.0001), and the presence of bilateral tumours (*p* < 0.0001) were associated with an increased risk of LNM. Variables with *p* < 0.2 in the univariate analysis results were further subjected to multivariate regression analysis. CT-reported LNM (OR: 7.86, 95% CI: 3.58, 17.28, *p* < 0.0001), nulliparity (OR: 0.30, 95% CI: 0.09, 1.04, *p* = 0.058), tumour bilaterality (OR: 1.90, 95% CI: 0.96, 3.78, *p* = 0.066), and advanced cFigo (OR: 1.84, 95% CI: 0.91, 3.73, *p* = 0.088) were found to be related to LNM (Figure 1). Although the CA125 concentration (OR: 4.07, 95% CI: 0.43, 38.62, *p* = 0.221) was non-significant, we assessed its complementary predictive value.

### 3.3. Nomogram Development and Evaluation

**Figure 2:**
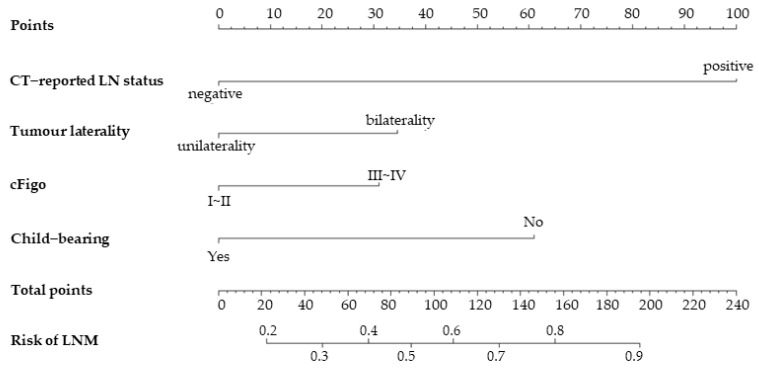
A nomogram combining the CT-reported LN status and clinical variables to identify patients with epithelial ovarian cancer having a high risk of LNM. Abbreviations: CT, computed tomography; LN, lymph node; cFigo, clinical International Federation of Obstetrics and Gynaecology.

Using preoperative variables (*p* < 0.1) in multivariate analysis, a nomogram was constructed to calculate the probability of LNM (Figure 2). The scores for each variable were plotted and then summed up to obtain a total score to assign the possibility of preoperative LNM.

**Figure 3:**
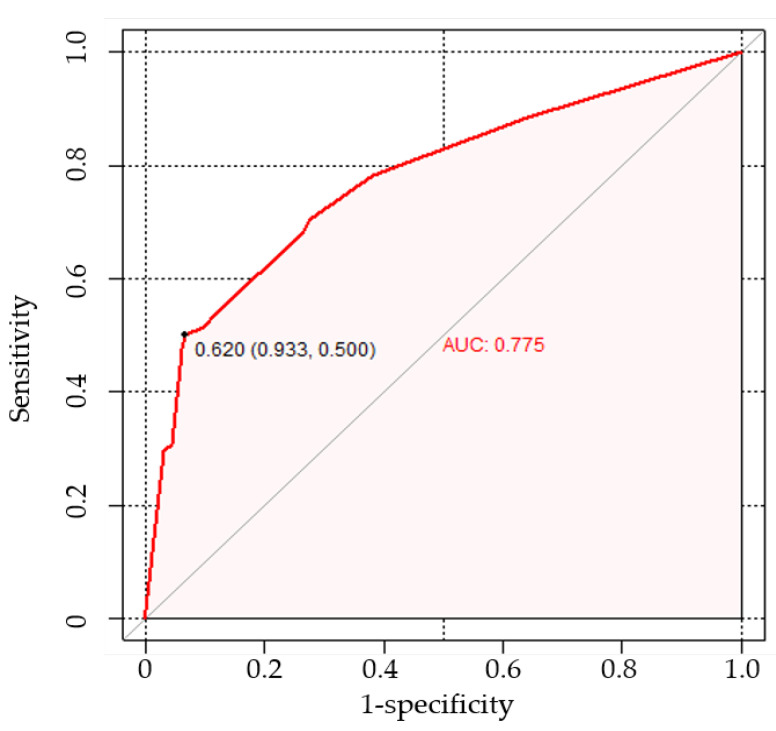
Receiver operating characteristic curve of the nomogram indicating an area under the curve of 0.775 (95% confidence interval, 0.713–0.829) in predicting LNM in patients with epithelial ovarian cancer. Abbreviations: LNM, lymph node metastasis.

**Figure 4:**
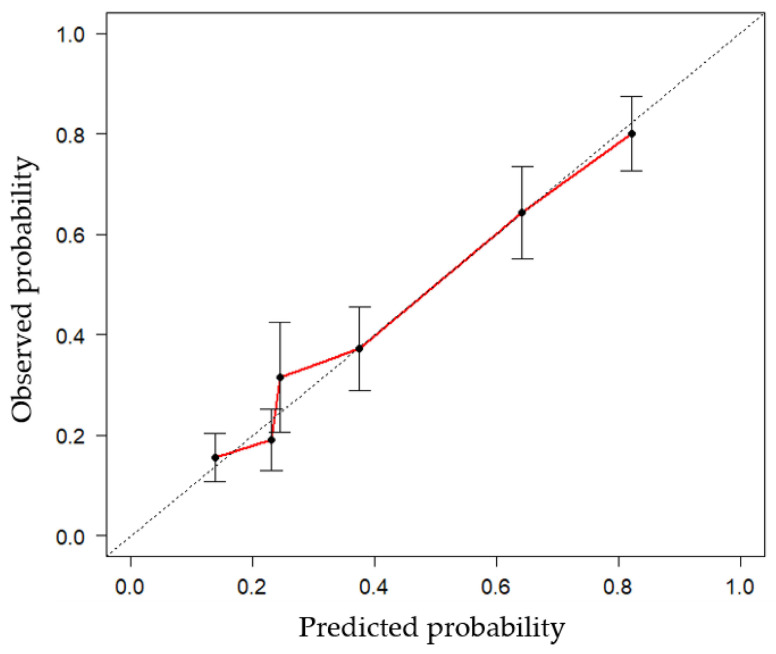
The plotted calibration curve shows good agreement between the predictive probability of the nomogram and the actual LN status. The red line indicates the performance of the nomogram, whereas the black dashed line indicates the actual LN status, as confirmed by the pathology results. Abbreviations: LN, lymph node.

**Figure 5:**
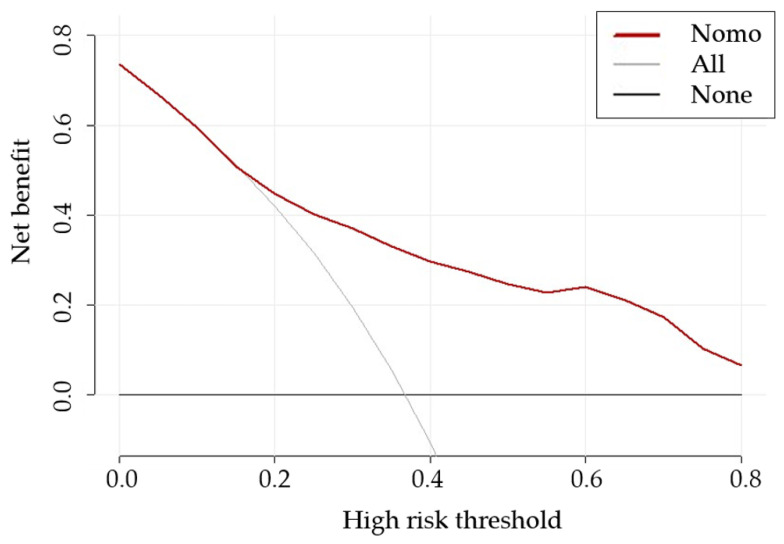
Decision curve analysis to assess the clinical utility of the proposed nomogram. The grey (All) and black (None) lines indicate the assumptions of all of the patients with and without LNM, respectively. The red line represents the performance of the nomogram and shows that the net clinical benefits can be achieved over a wide range of threshold possibilities. Abbreviations: LNM, lymph node metastasis; nomo, nomogram.

The AUC of the proposed nomogram was 0.775 (95% CI: 0.713, 0.829), highlighting its potential in assessing the risk of LNM (Figure 3). The calibration curve of the proposed nomogram demonstrated high consistency between the observed and predicted possibilities of the positive LNs (*p* = 0.999 in the Hosmer–Lemeshow test) (Figure 4). The mean squared error of prediction (Brier score) was only 0.172. The DCA performed to assess the clinical applicability of the risk prediction nomogram indicated a net benefit superior to those of the ‘all-lymphadenectomy’ and ‘non-lymphadenectomy’ strategies, with a wide range of threshold probabilities (Figure 5). 

### 3.4. Incremental Predictive Value of CA125 to the Nomogram

The nomogram with CA125 demonstrated good consistency between the predicted ability and actual status of LNM (*p* = 0.998, Brier score = 0.172). Although the nomogram with CA125 exhibited a slightly better AUC than that without CA125 (0.781 versus 0.775, *p* = 0.175), the difference was not significant (IDI = 0.003 (95% CI: −0.007 to −0.014), *p* = 0.552). An induction in event NRI (−4.13 (95% CI: −0.651 to −0.175), *p* < 0.001) indicated a net worsening in the prediction of LNM in patients with EOC.

### 3.5. Comparison with CT-Reported LN Status 

The CT-reported LNM and the nomogram were compared to further confirm the utility and predictive value of the nomogram. The AUC of the nomogram (0.775) was significantly greater than that of the CT scans (*p* = 0.002, 0.699 (95%CI: 0.632, 0.760)). The nomogram, which contained additional clinical variables, significantly improved the performance of image examinations with an NRI of 0.593 (95% CI: 0.331, 0.855, *p* < 0.001) and an IDI of 0.054 (95% CI: 0.024–0.085, *p* < 0.001). 

## 4. Discussion

In this retrospective study, we developed and internally validated a clinical variable-based, non-invasive nomogram integrating CT results and other clinical variables; the nomogram showed an AUC of 0.775 in identifying patients with suspicious LNs. This easy-to-use tool may help to avoid complications associated with unnecessary lymphadenectomy and determine optimal candidates for systemic lymphadenectomy. 

Systemic lymphadenectomy was advocated for its therapeutic and staging role. Prospective, randomised clinical trials provided the rational that optimal staging in early-stage disease was associated with significant improvements in the overall and recurrence-free survival of the patients, for reducing the possibility of micrometastases [30,31]. Lymphadenectomy in patients with advanced EOC was also associated with improved survival [32]. Survival benefit associated with lymphadenectomy was verified in a series of retrospective analysis [33,34,35]. However, other studies got contradictory results and found that lymphadenectomy did not bring more favourable outcomes, compared with those with no lymphadenectomy or bulky node resection only [36,37,38]. These studies were limited by their selection bias, small sample size, and the lack of surgery quality control. The prospectively randomised, well-designed, international, multicentre LION trial revealed pathology-confirmed LNM in 55.7% of patients from a lymphadenectomy group with a median of 57 resected LNs [7]. Improved outcomes were not observed in this group compared with the group without lymphadenectomy; rather, it caused a higher incidence of postoperative complications, providing strong evidence that lymphadenectomy was not a necessity in patients with clinically negative nodes. Later, suspicious and/or enlarged node resection was recommended in patients with extensive disease outside of the pelvis (>2 cm) by the National Comprehensive Cancer Network Clinical Practice Guideline [39]. For patients with stage I disease, single-agent adjuvant carboplatin can be used to reduce side effects of combination therapy [40]; and systemic lymphadenectomy was still recommended in patients with disease confined to the ovaries or the pelvis or tumour nodules outside the pelvis (≤2 cm). However, the rate of LNM is 3–14% among those with early-stage disease, suggesting a high possibility of overtreatment in patients with no LN involvement [8,10]. In other words, an actual preoperative evaluation of the LN status will benefit those with no LNM, and lymphadenectomy can thus be safely omitted.

Previous studies have investigated the risk factors associated with LNM. The histological tumour types, tumour grade, peritoneal washings, tumour laterality, CA125 concentrations, menopause status, and image examinations were reported to be associated with LN status. However, results of those studies varied owing to the different inclusion criteria and sample sizes [8,41,42,43,44]. In this study, we used the CT-reported LN status, child-bearing status, tumour laterality, and cFigo to construct a nomogram to identify patients with a high risk of LN involvement. 

The first strength of our study is that we used the pathology results as a reference standard and accordingly enrolled patients who had undergone both pelvic and para-aortic lymphadenectomy with no less than 10 nodes removed. The average number of LNs resected was 37.2 to reduce the chances of false negatives, which was higher than that reported in some previous studies [6,17,45]. Besides, we found a relatively higher prevalence of para-aortic LNM (31.6%) than pelvic LNM (25.9%) in our study; this finding corroborated those reported in previous studies, suggesting that the para-aortic area is the most common site of LNM [10,46]. 

Second, we only included preoperative parameters in the nomogram. The incidence of serous cystadenocarcinoma was the highest (28.0–36.7%), followed by endometrial cancer or clear cell carcinoma, and mucinous cancer rarely spread to the LNs [24,47]. Here, we obtained similar results and found that patients with high-grade serous cancer had an increased frequency of LNM compared with those with non-high-grade serous cancer (*p* = 0.0007). Positive peritoneal washings were also reported to be positively correlated with LNM (*p* = 0.035) [48]. However, such pathological information can only be obtained after surgery. In the multivariate analysis, women who had at least one child had a higher risk of LNM (*p* = 0.058). To the best of our knowledge, the role of parity has not yet been investigated in LNM in patients with EOC. The association between parity and prognosis in ovarian cancer remains a subject of debate. Some studies have suggested that high parity acts as a protective factor and is an indicator of less aggressive ovarian cancer and favourable outcomes, whereas others found no such significant associations [49,50,51]. The underlying mechanism is still unclear and needs further investigation. CA125, a high-molecular-weight mucinous glycoprotein present on the surface of ovarian cancer cells, acted as a predictive factor of LNM despite the different cut-off values used [20,44,45]. Ayhan et al. found that increasing CA125 concentrations indicated a larger number of metastatic LNs, suggesting that the CA125 concentration is correlated with intraabdominal and retroperitoneal tumour burden and metastasis [52]. The observation that the addition of CA125 did not improve the reclassification performance of the nomogram (NRI: −4.13) was unexpected; this might be attributable to the limited sample size and different cut-off values in the current study. 

Third, by adding clinical variables, the performance of the CT results was significantly boosted, with an NRI of 0.593 and an IDI of 0.054. CT, the most frequently used imaging technique in the preoperative evaluation of ovarian cancer, achieved an AUC of 0.699, which was similar to that reported in other studies [18,19]. A short-axis diameter threshold of 10 mm failed to consider normal-sized LNs with microscopic or partial infiltration or inflammatory node enlargement, leading to misdiagnosis as well as missed diagnosis. A decision for or against lymphadenectomy may not be made solely on the basis of CT results. ^18^FDG positron emission tomography achieved a higher diagnostic accuracy than CT in revealing the metabolic or biochemical information of patients with ovarian cancer, even of those with normal-sized metastatic LNs [14]; this will be further investigated in future studies. 

We acknowledge that there are several limitations to this study. First, this retrospective study was conducted at a single centre with a small sample size. A larger sample size and independent external evaluations are thus needed in further studies to verify our findings. Second, because the patients who underwent neoadjuvant chemotherapy were excluded from the model development, caution is needed before generalising our nomogram to such patients. Third, the results of the image examination contributed the most to the nomogram, although interobserver agreement across centres could bring heterogeneity in real-world settings. Multicentre studies are thus warranted to confirm the effectiveness of the nomogram.

## 5. Conclusions

To conclude, we developed and internally validated a clinical parameter-based nomogram that allows a more accurate stratification of patients with EOC into low-risk or high-risk groups of LNM than conventional imaging examinations. This easy-to-use tool has the potential to help clinicians to identify candidates for systemic lymphadenectomy and avoid unnecessary surgeries in the precision medicine era of personalized care, which might serve as a clinical applicable tool after extensive clinical validation.

## Figures and Tables

**Table 1:** Baseline characteristics of the patients included in this study.

	All (n = 212)	LNM Negative (n = 134)	LNM Positive (n = 78)
Age			
Mean ± SD	50.8 (9.4)	50.9 (9.3)	50.7 (9.7)
Range	27–75	27–75	28–75
Child-bearing			
No	13 (6.1)	6 (4.5)	7 (9.0)
Yes	199 (93.9)	128 (95.5)	71 (91.0)
Histological type			
High-grade serous cancer	124 (58.5)	69 (51.5)	55 (70.5)
Clear cell carcinoma	34 (16.0)	25 (18.7)	9 (11.5)
Endometrial cancer	25 (11.8)	23 (17.2)	2 (2.6)
Low-grade serous cancer	9 (4.2)	5 (3.7)	4 (5.1)
Mixed carcinoma	5 (2.4)	3 (2.2)	2 (2.6)
Other	15 (7.1)	9 (6.7)	6 (7.7)
Pelvic LNM			
Negative	157 (74.1)	134 (100)	23 (29.5)
Positive	55 (25.9)	0 (0.0)	55 (70.5)
Para-aortic LNM			
Negative	145 (68.4)	134 (100)	11 (14.1)
Positive	67 (31.6)	0 (0.0)	67 (85.9)
Peritoneal washings			
Negative	94 (44.3)	66 (49.3)	28 (35.9)
Positive	101 (47.6)	60 (44.8)	41 (52.6)
Unknown	17 (8. 2)	8 (5.9)	9 (11.5)
CT-reported LN status			
Negative	162 (76.4)	122 (91.0)	40 (51.3)
Positive	50 (23.6)	12 (9.0)	38 (48.7)
cFigo			
I–II	89 (42.0)	70 (52.2)	19 (24.4)
III–IV	123 (58.0)	64 (47.8)	59 (75.6)
Laterality			
Unilaterality	122 (57.5)	87 (64.9)	35 (44.9)
Bilaterality	90 (42.5)	47 (35.1)	43 (55.1)
Ascites			
Negative	87 (41.0)	55 (41.0)	32 (41.0)
Positive	125 (59.0)	79 (59.0)	46 (59.0)
CA125			
<35 U/mL	13 (6.1)	12 (9.0)	1 (1.3)
≥35 U/mL	199 (93.9)	122 (91.0)	77 (98.7)
CA153			
<25 U/mL	73 (34.4)	51 (38.1)	22 (28.2)
≥25 U/mL	139 (65.6)	83 (61.9)	56 (71.8)
CA199			
<35 U/mL	135 (63.7)	86 (64.2)	49 (62.8)
≥35 U/mL	77 (36.3)	48 (35.8)	29 (37.2)

Abbreviations: LNM, lymph node metastasis; SD, standard deviation; CT, computed tomography; LN, lymph node; cFigo, clinical International Federation of Obstetrics and Gynaecology; CA, cancer antigen.

**Table 2:** Univariate and multivariate regression analyses of clinical factors associated with LNM in patients with epithelial ovarian cancer.

	Univariate Analysis			Multivariate Analysis		
	OR	95% CI	*p*	OR	95% CI	*p*
Age	1.00	(0.97–1.03)	0.905			
Child-bearing	0.48	(0.15–1.47)	0.197	0.30	(0.09–1.04)	0.058
Ascites	1.00	(0.57–1.77)	0.998			
Tumour laterality	2.89	(1.62–5.16)	<0.0001	0.53	(0.27–1.04)	0.066
cFigo	3.4	(1.83–6.30)	<0.0001	1.84	(0.91–3.73)	0.088
CT-reported LN status	9.66	(4.61–20.26)	<0.0001	7.86	(3.58–17.28)	<0.0001
CA125	7.57	(0.97–59.42)	0.054	4.07	(0.43–38.62)	0.221
CA153	1.56	(0.86–2.86)	0.147	1.02	(0.48–2.17)	0.953
CA199	1.06	(0.59–1.89)	0.843			

Abbreviations: LNM, lymph node metastasis; CT, computed tomography; cFigo, clinical International Federation of Obstetrics and Gynaecology; OR, odds ratio; CI, confidence interval.

## Data Availability

The private data analysed in this study are not publicly available due to hospital regulations and patient privacy considerations. All data generated or analysed during the study are included in the published paper. However, some data can be made available from the corresponding author on reasonable request only for academic purposes.

## Supplementary Materials

The following supporting information can be downloaded at: https://www.mdpi.com/article/10.3390/curroncol30030250/s1, Table S1: CT scanners and parameters.
