# Supplementary material for: A Nomogram for Preoperative Prediction of the Risk of Lymph Node Metastasis in Patients with Epithelial Ovarian Cancer
Source: Curr Oncol. 2023 Mar 13;30(3):3289–300. doi: 10.3390/curroncol30030250 (PMC10047242; doi:10.3390/curroncol30030250)
Supplement: Supplementary file 1 [file curroncol-30-00250-s001.zip › curroncol-2226852-supplementary.pdf]

Table S1. CT scanners and parameters.

CT scanners	Aquilion IQon Spectral CT	Discovery 750 HD	Somatom Force	uCT780
	**Philips Healthcare, Best, The Netherlands**	**GE Healthcare, Milwaukee, WI**	**Siemens Healthcare GmbH, Erlangen, Germany**	**United Imaging Healthcare, Shanghai, China**
tube voltage (kVp)	120	120	120	120
tube current(mA)	252-426	171-348	134-314	134-314
slice thickness(mm)	5	5	5	5
slice interval(mm)	5	5	5	5
Matrix	512*512	512*512	512*512	512*512

Abbreviations: CT, computed tomography.
